# CD20+ T cells in monoclonal B cell lymphocytosis and chronic lymphocytic leukemia: frequency, phenotype and association with disease progression

**DOI:** 10.3389/fonc.2024.1380648

**Published:** 2024-03-28

**Authors:** Cristiana Rodrigues, Paula Laranjeira, Aryane Pinho, Isabel Silva, Sandra Silva, Margarida Coucelo, Ana Catarina Oliveira, Ana Teresa Simões, Inês Damásio, Helena Matos Silva, Mafalda Urbano, Ana Bela Sarmento-Ribeiro, Catarina Geraldes, M. Rosário Domingues, Julia Almeida, Ignacio Criado, Alberto Orfao, Artur Paiva

**Affiliations:** ^1^ Flow Cytometry Unit, Department of Clinical Pathology, Centro Hospitalar e Universitário de Coimbra, Coimbra, Portugal; ^2^ Department of Chemistry, University of Aveiro, Aveiro, Portugal; ^3^ Coimbra Institute for Clinical and Biomedical Research (iCBR), Group of Environmental Genetics of Oncobiology (CIMAGO), Faculty of Medicine (FMUC), University of Coimbra, Coimbra, Portugal; ^4^ Center for Innovative Biomedicine and Biotechnology (CIBB), University of Coimbra, Coimbra, Portugal; ^5^ Clinical Academic Center of Coimbra (CACC), Coimbra, Portugal; ^6^ Center for Neuroscience and Cell Biology (CNC), University of Coimbra, Coimbra, Portugal; ^7^ Department of Life Sciences, Faculty of Sciences and Technology, University of Coimbra, Coimbra, Portugal; ^8^ Unidade Funcional de Hematologia Molecular, Serviço de Hematologia Clínica, Centro Hospitalar e Universitário de Coimbra, Coimbra, Portugal; ^9^ Hematology Department, Centro Hospitalar Tondela-Viseu, Viseu, Portugal; ^10^ Hematology Department, Centro Hospitalar e Universitário de Coimbra, Coimbra, Portugal; ^11^ University Clinics of Hematology and Oncology and Laboratory of Oncobiology and Hematology, Faculty of Medicine, University of Coimbra, Coimbra, Portugal; ^12^ Mass Spectrometry Centre, Associated Laboratory for Green Chemistry (LAQV-REQUIMTE), Department of Chemistry, University of Aveiro, Aveiro, Portugal; ^13^ CESAM—Centre for Environmental and Marine Studies, Department of Chemistry, University of Aveiro, Aveiro, Portugal; ^14^ Translational and Clinical Research Program, Cancer Research Center (IBMCC, CSIC-University of Salamanca), Salamanca, Spain; ^15^ Department of Medicine, University of Salamanca (Universidad de Salamanca), Salamanca, Spain; ^16^ Institute of Biomedical Research of Salamanca (IBSAL), Salamanca, Spain; ^17^ Biomedical Research Networking Centre Consortium of Oncology (CIBERONC), Instituto de Salud Carlos III, Madrid, Spain; ^18^ Ciências Biomédicas Laboratoriais, Instituto Politécnico de Coimbra, Escola Superior de Tecnologia da Saúde de Coimbra (ESTESC)-Coimbra Health School, Coimbra, Portugal

**Keywords:** CD20+ T cells, chronic lymphocytic leukemia, monoclonal B lymphocytosis, immunoglobulin heavy chain variable region, T cell polarization

## Abstract

**Introduction:**

In monoclonal B cell lymphocytosis (MBL) and chronic lymphocytic leukemia (CLL), the expansion of malignant B cells disrupts the normal homeostasis and interactions between B cells and T cells, leading to immune dysregulation. CD20+ T cells are a subpopulation of T cells that appear to be involved in autoimmune diseases and cancer.

**Methods:**

Here, we quantified and phenotypically characterized CD20+ T cells from MBL subjects and CLL patients using flow cytometry and correlated our findings with the B-cell receptor mutational status and other features of the disease.

**Results and discussion:**

CD20+ T cells were more represented within the CD8+ T cell compartment and they showed a predominant memory Tc1 phenotype. CD20+ T cells were less represented in MBL and CLL patients vs healthy controls, particularly among those with unmutated IGVH gene. The expansion of malignant B cells was accompanied by phenotypic and functional changes in CD20+ T cells, including an increase in follicular helper CD4+ CD20+ T cells and CD20+ Tc1 cells, in addition to the expansion of the TCR Vβ 5.1 in CD4+ CD20+ T cells in CLL.

## 1 Introduction

Monoclonal B lymphocytosis (MBL) and chronic lymphocytic leukemia (CLL) are both characterized by the accumulation of clonal B lymphocytes with a mature phenotype and an abnormal BCR signaling and function in peripheral blood, bone marrow or secondary lymphoid tissues (1, 2). CLL-like MBL shares immunophenotypic, biological, and molecular features with CLL, its distinction from CLL being mostly based on the monoclonal B lymphocyte count (< 5000 cells/μL) in the absence of signs or symptoms of a B cell lymphoproliferative disorder (1, 3, 4). Based on the clonal B cell count, MBL can be further categorized as low-count (LC) (<500 cells/μL) or high-count (HC) MBL (>500 cells/μL). HC-MBL is thought to be a pre-malignant state that precedes CLL and it is associated with an annual rate of progression to CLL of 1-2% (3–6). Up to now, a few tumor cell features have been associated with progression from MBL to CLL, including an increase in total lymphocyte count in blood and an unmutated somatic hypermutation (SHM) status of the immunoglobulin heavy chain variable region (IGHV) gene (3, 6–9).

For decades now, it is well known that the expansion of malignant B cells is accompanied by numerical and phenotypic changes in T cells, including clonal T cell expansions, with preferential usage of restricted TCR Vβ gene families, a higher frequency of regulatory T cells (Tregs), reduced naïve CD4+ T cell counts and an enrichment in memory CD4+ T cells, together with a higher frequency of follicular, Th1, Th17, and CD8+ T cells. In addition, functional exhaustion of CD8+ T cells has been claimed to be responsible for an impaired cytotoxic response against tumor cells, which might favor the growth and expansion of malignant B cells (3, 10–14).

Recently, a new subpopulation of T cells, characterized by the expression of CD20 (a membrane protein expressed by mature B cells) has been identified (15). The origin of CD20+ T cells remains uncertain, trogocytosis being identified as one of the potential mechanisms for the acquisition of this B-cell associated receptor by T cells, though the presence of CD20 mRNA in these cells contradicts this hypothesis (15–18). From the functional point of view, CD20+ T cells have been reported to be involved in the pathophysiology of autoimmune diseases, particularly in multiple sclerosis, as well as in cancer, where numerical, phenotypic and functional alterations of these cells have been detected (18–24). Studies in autoimmune diseases suggest a pathogenic behavior of CD20+ T cells by producing IL-17 and TNF-α (17, 20, 22, 25). In contrast, other studies suggest a protective role for these cells in cancer. Independently of their specific pathogenic role, CD20+ T cells exhibit a predominantly Tc1 effector memory phenotype, suggesting an antitumor activity (15, 18, 24, 25).

This study aims to contribute to a better understanding of the role of CD20+ T cells, in LC-MBL, HC-MBL and CLL, through their enumeration and both phenotypic and functional characterization in these conditions, compared to a group of age-matched healthy subjects.

## 2 Materials and methods

### 2.1 Study population

In the present study, peripheral blood samples from 13 subjects with confirmed diagnosis of LC-MBL (8 men and 5 women; mean age: 68 ± 9 years), 12 subjects with HC-MBL (8 men and 4 women, mean age: 74 ± 12 years) and 27 patients newly-diagnosed with CLL (21 men and 6 women; mean age: 74 ± 11 years) were collected in EDTA, processed and analyzed by flow cytometry, to quantify and phenotypically characterize blood circulating CD20+ T cells. In addition, 10 peripheral blood samples from sex- and age-matched healthy controls (8 men and 2 women; mean age: 58 ± 3 years), were also studied in parallel, after confirming the absence of MBL. In a subset of 5 CLL patients (2 men and 3 women; mean age: 67 ± 11 years) and 5 (non-MBL) healthy controls (3 men and 2 women; mean age: 75 ± 8 years) analysis of the TCR Vβ repertoire on peripheral blood CD4+ CD20-, CD8+CD20-, CD4+CD20+ and CD8+CD20+ T cells was also performed.

### 2.2 Phenotypic analysis of CD20+ T cells by flow cytometry

#### 2.2.1 Staining protocol and sample acquisition

Processing of samples for phenotypic analysis of CD20+ T cells was performed following the EuroFlow Standard Operating Procedure (SOP) for sample preparation and staining, and acquisition in a flow cytometer (26). Briefly, 100 µL of the peripheral blood was transferred to a test tube which already contained the monoclonal antibodies described in detail in **Table 1**. Afterwards, a previously described Lyse-Wash protocol (26) was followed. Finally, the cell pellet was resuspended in PBS and immediately acquired in a FACSLyric flow cytometer (Becton Dickinson Biosciences [BD], San Jose, CA, USA), equipped with the FACSuite software (v1.5.0.925; BD).

**Table 1:** Monoclonal antibody panel used for identification and phenotypic characterization of CD20+ T cells.

Antibody	Fluorochrome	Clone	Commercial source
CD3	BV605	SK7	BD
CD4	PerCP-Cy 5.5	SK3	BD
CD8	APC-H7	SK1	BD
CD20	V450	L27	BD
CD25	FITC	2A3	BD
CD27	APC-R700	M-T271	BD
CD45RA	BV786	HI100	BD
CD127	BV510	HIL-7R-M21	BD
CD195	BV711	2D7/CCR5	BD
CD196	PE	11A9	BD
CXCR5	APC	51505	R&D Systems
TCR γδ	PE-Cy7	11F2	BD

APC, allophycocyanine; APC-H7, allophycocyanine-hilite 7; BV, brilliant violet; FITC, fluorescein isothiocyanate; PE, phycoerythrin; PE-Cy7, phycoerythrin-cyanine 7; PerCP-Cy5.5, peridinin chlorophyll protein cyanine 5.5.; BD: Becton Dickinson Biosciences, San Jose, CA, USA; R&D Systems: Minneapolis, MN, USA.

#### 2.2.2 Data analysis

Data analysis was performed using the Infinicyt 2.0.5 software (Cytognos SL, Salamanca, Spain). T cells were identified based on their positivity for CD3 and typically low side scatter (SSC) and forward scatter (FSC) properties. CD20 positivity was used to identify CD20+ T cells. Among these, CD4+ T cells were identified by their expression of CD4 and absence of CD8, while CD8+ T cells were identified by expression of CD8 in the absence of CD4. Double positive T cells (CD4+ CD8+) were identified by expression of both CD4 and CD8, and TCR γδ+ T cells were defined based on their positivity for TCR γδ, while TCR αβ+ CD4- CD8- T cells were identified by the expression of CD3 in the absence of CD4, CD8 and TCR γδ. According to CXCR5 expression, CXCR5+ follicular helper T cells and CXCR5- negative T cells were identified among CD4+ CD20+ T cells. Four additional subpopulations of CD4+ CD20+ follicular helper T (CXCR5+, TFH) cells were also identified based on the pattern of expression of CCR5 and CCR6: CCR5+ CCR6- (Th1-like), CCR5- CCR6+ (Th17-like), CCR5+ CCR6+ (Th1/17-like) and CCR5- CCR6- TFH cells. Th17 cells (CCR6+) and Th1 cells (CCR5+) were identified within CXCR5- CD4+ CD20+ T cells. Among CCR6+ cells, CCR5 positive (CCR6+ CCR5+) cells and CCR5 negative cells (CCR6+ CCR5-) were also identified.

**Figure 1:**
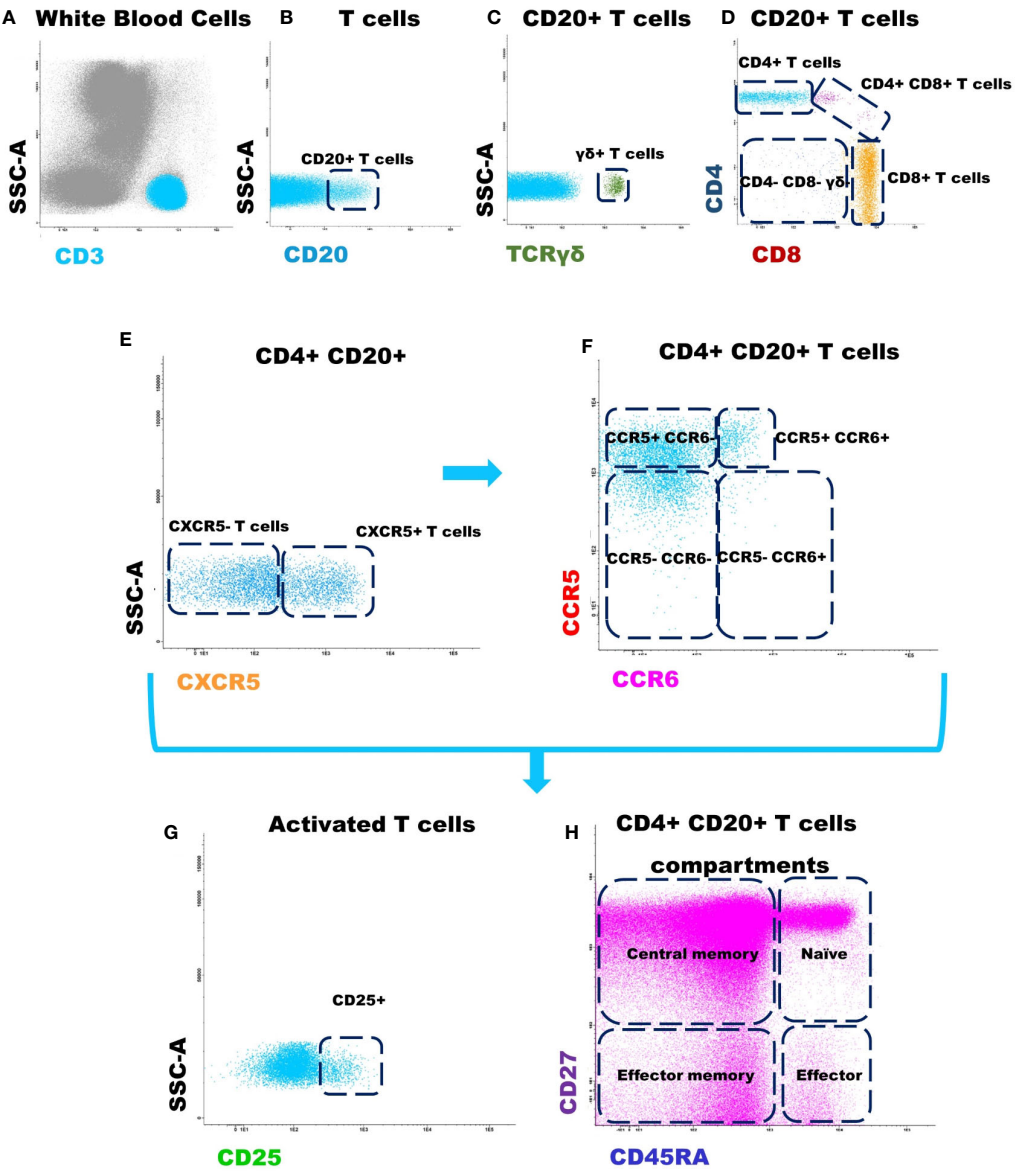
Gating strategy used for the identification and phenotypic characterization of CD20+ T cells. **(A)** T cells were identified based on CD3 positivity and typically low side scatter (SSC) and forward scatter (FSC) properties; **(B)** Identification of CD20+ T cells according to CD20 expression; **(C)** Identification of γδ+ T cells, among CD20+ T cells, according to TCR γδ expression; **(D)** Identification of CD4+, CD8+, CD4+/CD8+ and TCR αβ+ CD4- CD8- subsets within among CD20+ T cells; **(E)** CD4+ CD20+ follicular T cells were identified by positivity for CXCR5; **(F)** The subpopulations of T cells: CCR5+ CCR6- (Th1), CCR5- CCR6+ (Th17), CCR5+ CCR6+ (Th1/17), and CCR5- CCR6- T cells were identified within CXCR5+ CD4+ CD20+ follicular helper T cells and CXCR5- CD4+ CD20+ T cells, respectively. The same strategy was used for the other T cell subpopulations analyzed. **(G)** Dot plot histograms illustrating the identification of activated CD20+ T cells (based on CD25 expression); **(H)** CD45RA and CD27 were used to identify naïve (CD45RA+ CD27+), central memory (CD45RA- CD27+), effector memory (CD45RA- CD27-) and terminal effector (CD45RA+ CD27-) maturation-associated T cells compartments on the above mentioned subsets of CD20+ T cells.

The same strategy described to identify T cell subpopulations within CD4+ CD20+ cells was used for CD8+ CD20+ T cells. Finally, for each CD20+ T cell subset identified up to this point, the percentage of activated (CD25+) T cells and the distribution by maturation-associated compartments of CD20+ T cells: naïve (CD45RA+ CD27+), central memory (CD45RA- CD27+), effector memory (CD45RA- CD27-) and terminal effector (CD45RA+ CD27-) T cell compartments were also evaluated. The gating strategy used for the identification and phenotypic characterization of CD20+ T cells is illustrated in **Figure 1**.

### 2.3 Analysis of the T cell receptor (TCR)-Vβ repertoire by flow cytometry

The study of the TCR Vβ repertoire was performed with the IOTest Beta Mark TCR-Vβ Repertoire kit (Beckman Coulter - Immunotech, Marseille, France), which includes the following monoclonal antibodies: Vβ1 (clone BL37.2); Vβ2 (clone MPB2D5); Vβ3 (clone CH92); Vβ4 (clone WJF24); Vβ5.1 (clone IMMU157); Vβ5.2 (clone 36213); Vβ5.3 (clone 3D11); Vβ7.1 (clone ZOE); Vβ7.2 (clone ZIZOU4); Vβ8 (clone 56C5.2); Vβ9 (clone FIN9); Vβ11 (clone C21); Vβ12 (clone VER2.32.1.1); Vβ13.1 (clone IMMU222); Vβ13.2 (clone H132); Vβ13.6 (clone JU74.33); Vβ14 (clone CAS1.1.3); Vβ16 (clone TAMAYA1.2); Vβ17 (clone E17.5F3.15.13); Vβ18 (clone BA62.6); Vβ20 (clone ELL1.4); Vβ21.3 (clone IG125); Vβ22 (clone IMMU546) and Vβ23 (clone AF23). This kit contains 8 tubes, each with antibodies against 3 TCR-Vβ regions where the first antibody is conjugated with fluorescein isothiocyanate (FITC), the second with phycoerythrin (PE), and the third with both FITC and PE. Antibody labeling was performed by incubating 100 μL of peripheral blood with each TCR Vβ antibody mixtures (20 μL) for 20 minutes. After this incubation period, to evaluate the expression of TCR Vβ in CD4+ CD20+ and CD8+ CD20+ T cell subpopulations, the CD45-V500-C (clone 2D1, BD), CD3-PerCP-Cy 5.5 (clone SK7, BD), CD8-APC (clone SK1, BD), CD4-APC-H7 (clone SK3, BD) and CD20-PB (clone L27, BD) were also added to stain the peripheral blood sample. Then, a 10-minute incubation period at room temperature in the dark followed by a lysis and washing step protocol, was performed (26).

### 2.4 Cell purification

For each peripheral blood sample from CLL and HC-MBL patients, the CD5+ B cell population was purified on a FACSAria III (BD) flow cytometer. For this purpose, blood samples were labeled with CD45-V500-C (clone 2D1, BD); CD3-PB (clone UCHT1, BD); CD19-PC7 (clone J3-119, Beckman Coulter) and CD5-APC (clone L17F12, BD). The purity of the purified cell populations was always > 97%.

### 2.5 Molecular studies

Analysis of the TP53 gene and the IGHV gene mutational status was performed on DNA extracted from purified pathological B cells using an automated extraction system (QIAsymphony SP, Qiagen, Hilden, Germany) based on the manufacturer’s reagents and protocols. The search for mutations in the TP53 gene was performed by Next-Generation Sequencing (NGS) with the Ion AmpliSeq TP53 Panel (Thermo Fisher Scientific, Waltham, MA, USA) on the Ion S5 sequencer (Thermo Fisher Scientific). The analysis of the results was performed using the Ion Reporter (Thermo Fisher Scientific) and Integrative Genomics Viewer (IGV) (v2.16.2, UC San Diego, CA, USA; and Broad Institute of MIT Harvard, Boston, MA, USA) software. Determination of the IGHV mutational status was performed by PCR/fragment analysis and Sanger sequencing, according to the ERIC (European Research Initiative on CLL) recommendations. Sequences were analyzed in IMGT/V-QUEST (v1.9.3, LIMG, Montpellier, France), to identify clonal rearrangements and to calculate their percent identity to the germline gene sequence. According to the ERIC recommendations, sequences with a percentage of identity lower than 98% were considered to be mutated while those with a ≥ 98% identity were reported as unmutated (27).

### 2.6 Statistical analysis

The statistical significance of differences between groups was analyzed using the non-parametric Kruskal-Wallis and Mann–Whitney U tests for continuous variables, or the Pearson’s x2 and Fisher exact tests for categorical variables. Statistically significant differences were considered to be present when p values of less than 0.05 were detected with the GraphPad Prism 8.4.0 software (San Diego, CA, USA).

## 3 Results

### 3.1 Numerical distribution of CD20+ T cells in blood of MBL and CLL vs controls

In healthy individuals, the majority of CD20+ T cells belonged to the CD8+ compartment of T cells, followed by the CD4+, CD4+ CD8+ and TCR γδ + compartments, while they were almost absent in the TCR αβ+ CD4- CD8- T cells (**Table 2**).

**Table 2:** Distribution of CD20+ T cells (mean ± standard deviation) in blood of LC-MBL, HC-MBL and CLL subjects compared to non-MBL healthy controls.

	Controls (n = 10)	LC-MBL (n = 13)	HC-MBL (n = 12)	CLL (n = 27)
**CD20+ T cells** No. of cells/µL % of leukocytes % ​​after excluding neoplastic B cells	60 ± 44 0.98 ± 0.60	34 ± 32 0.35 ± 0.26^*a*^ 0.35 ± 0.26^*a*^	38 ± 30 0.42 ± 0.26^*a*^ 0.58 ± 0.42	33 ± 40 ^*a*^ 0.14 ± 0.17 ^*a b c*^ 0.43 ± 0.47 ^*a*^
% CD20+ T cells	4.7 ± 2.0	2.6 ± 1.4	3.5 ± 2.1	2.8 ± 2.7 ^*a*^
% CD20+ CD4+ T cells	31 ± 13	31 ± 19	22 ± 11	31 ± 20
% CD20+ CD8+ T cells	58 ± 15	65 ± 18	74 ± 14	61 ± 24
% CD20+ CD4+ CD8+ T cells	6.5 ± 11	0.88 ± 1.0	0.90 ± 1.4	0.62 ± 1.3 ^*a b*^
% CD20+ TCR γδ+ T cells	3.0 ± 3.3	2.1 ± 1.9	2.9 ± 5.7	1.3 ± 2.0
% CD20+ TCR αβ+ CD4- CD8- TCR γδ- T cells	0.51 ± 0.74	0.94 ± 1.2	0.71 ± 1.1	5.1 ± 13

a p<0.05 vs controls; ^b^p<0.05 vs LC-MBL; ^c^p<0.05 vs HC-MBL. CLL, chronic lymphocytic leukemia; HC, high-count; LC, low-count; MBL, monoclonal B lymphocytosis.

**Figure 2:**
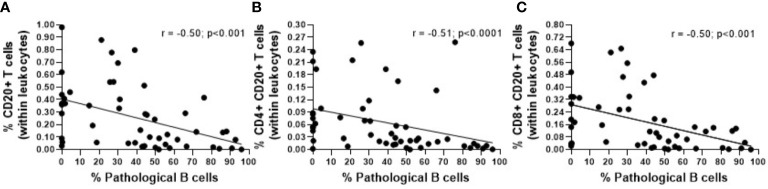
Correlation between the percentage of pathological B cells and the percentage of **(A)** CD20+ T cells (within leukocytes), **(B)** CD4+ CD20+ T cells (within leukocytes) and **(C)** CD8+ CD20+ T cells (within leukocytes). The r and p values ​​were determined according to Spearman’s correlation.

Overall, a statistically significant decrease in the absolute count of CD20+ T cells was observed in CLL compared to non-MBL healthy individuals, the percentages of CD20+ T cells being significant lower in both LC-MBL and CLL compared to the control group (**Table 2**). This decrease inversely correlated with the expansion of pathological B cells, as shown in **Figure 2**. Of note, in both MBL cases and CLL patients, the percentage of CD20+ T cells was higher among TCR γδ+ T cells than T CD4+ CD8+ cells (**Table 2**). Interestingly, HC-MBL subjects displayed a decrease in the percentage of CD20+ CD4+ T cells and an increase in CD20+ CD8+ T cells compared to the other groups, with a statistically significant difference between HC-MBL and the control group (p<0.05 vs controls) (**Table 2**).

### 3.2 Phenotypic characterization of CD20+ T cells in blood of MBL and CLL vs controls

Since CD20+ T cells are more represented in both the CD8+ and CD4+ T cell compartments, we focused on those T cell populations to perform a more in depth phenotypic characterization of CD20+ T cells (**Supplementary Figure 1**).

#### 3.2.1 CD4+ CD20+ T cells

**Figure 3:**
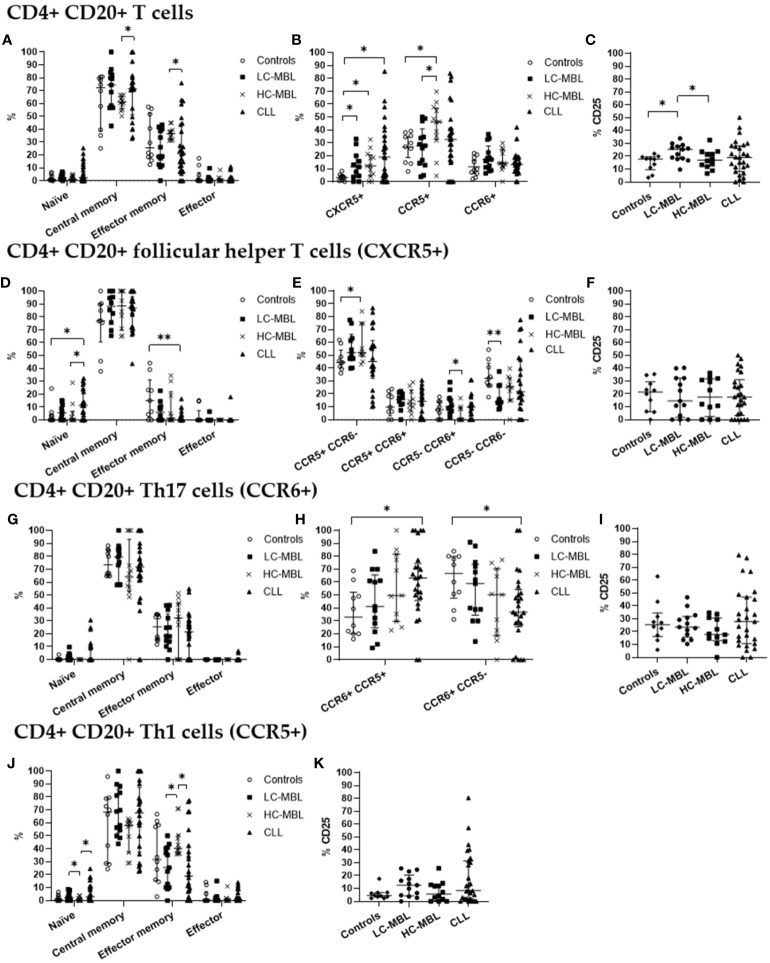
Phenotypic characterization of CD4+ CD20+ T cells and their subsets in blood of LC-MBL, HC-MBL and CLL vs controls. **(A-C)** Analysis of total CD4+ CD20+ T cells: **(A)** distribution among each different maturation-associated compartment of CD20+ T cells (naïve, central memory, effector memory and terminal effector); **(B)** percentage of CD4+ follicular helper T (TFH) cells (CXCR5+), Th1 (CCR5+) and Th17 (CCR6+) cells; and **(C)** percentage of activated (CD25+) CD4+ CD20+ T cells. **(D-F)** Distribution of CD4+ CD20+ TFH cells in **(D)** the different maturation-associated T cell compartments (naive, central memory, effector memory and terminal effector); and **(E)** TFH cell subsets (TFH1, TFH17, TFH1/17 and CCR5- CCR6- TFH cells). **(F)** Percentage of activated (CD25+) TFH cells. **(G-I)** Analysis of CD20+ Th17 cells: **(G)** frequency of cells in the different maturation-associated compartments of CD20+ Th17 cells (naïve, central memory, effector memory and terminal effector; **(H)** percentage of CD20+ Th17 cells CCR5+ or CCR5-; **(I)** percentage of activated (CD25+) CD20+ Th17 cells. **(J)** Frequency of CD20+ Th1 cells within each different maturation-associated compartments of CD20+ T cells: naïve, central memory, effector memory and terminal effector. **(K)** Percentage of activated (CD25+) CD20+ Th1 cells. Data expressed as median and interquartile range. *p < 0.05; **p < 0.01. CLL, chronic lymphocytic leukemia; HC, high-count; LC, low-count; MBL, monoclonal B lymphocytosis.

**Figure 4:**
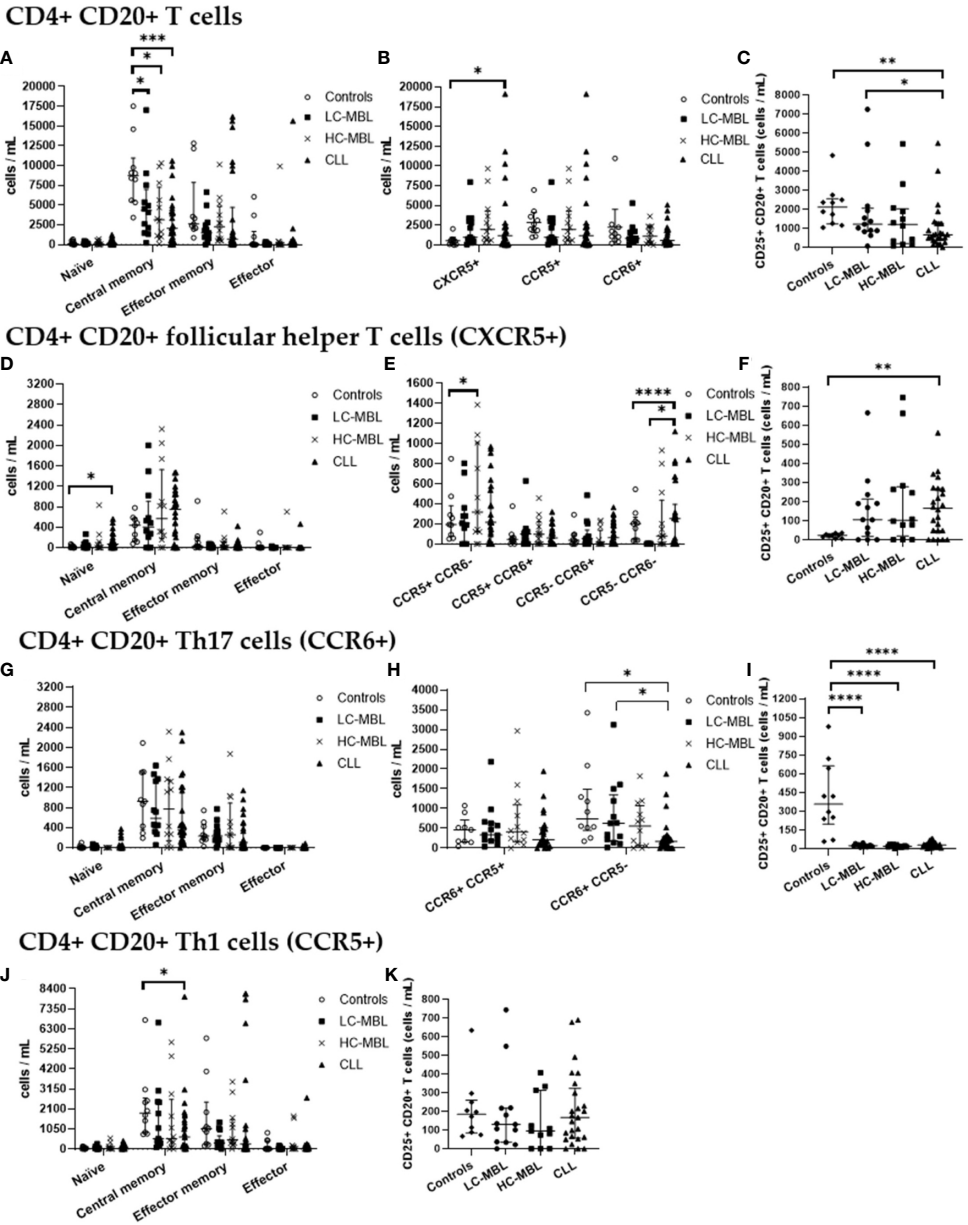
Absolute number of CD4+ CD20+ T cells and their subsets in blood of LC-MBL, HC-MBL and CLL vs controls. **(A-C)** Analysis of total CD4+ CD20+ T cells: **(A)** distribution within each different maturation-associated compartment of CD20+ T cells (naïve, central memory, effector memory and terminal effector); **(B)** absolute number of CD4+ follicular helper T (TFH) cells (CXCR5+), Th1 (CCR5+) and Th17 (CCR6+) cells; and **(C)** absolute number of activated (CD25+) CD4+ CD20+ T cells. **(D-F)** Distribution of CD4+ CD20+ TFH cells in **(D)** different T cell compartments (naive, central memory, effector memory and terminal effector); **(E)** TFH cell subsets (TFH1, TFH17, TFH1/17 and CCR5- CCR6- TFH cells). **(F)** absolute number of activated (CD25+) TFH cells. **(G-I)** Analysis of CD20+ Th17 cells: **(G)** absolute number of cells in the different maturation-associated compartments of CD20+ T cells (naïve, central memory, effector memory and terminal effector); **(H)** absolute number of CD20+ Th17 cells CCR5+ or CCR5-; **(I)** absolute number of activated (CD25+) CD20+ Th17 cells. **(J)** Absolute number of CD20+ Th1 cells within each different maturation-associated compartments of CD20+ T cells: naïve, central memory, effector memory and terminal effector; and **(K)** absolute number of activated (CD25+) CD20+ Th1 cells. Data expressed as median and interquartile range. *p < 0.05; **p < 0.01; ***p < 0.001; ****p < 0.0001. CLL, chronic lymphocytic leukemia; HC, high-count; LC, low-count; MBL, monoclonal B lymphocytosis.

In healthy individuals, CD4+ CD20+ T cells showed a predominant central and effector memory phenotype (**Figures 3A**, **4A**); (**Supplementary Figures 2**, **3**). This memory phenotype predominated among all functional subpopulations of CD4+ CD20+ T cells including Th1, Th17 and TFH cells (**Figures 3D, G, J**, **4D, G, J**, respectively). Furthermore, we also observed that in healthy individuals, the majority of CD4+ CD20 TFH cells displayed a Th1-like phenotype, while the majority of CD4+ CD20+ Th17 cells were CCR5- (**Figures 3E, H**, **4H**).

From the maturation point of view, there was a statistically significant decrease in the absolute number of central memory cells across all patient groups when compared to the control group (**Figure 4A**). Of note, the percentage of central memory cells increased, and the relative numbers of effector memory cells was significantly lower in CLL than HC-MBL (**Figure 3A**).

Moreover, a statistically significant increase in the percentage and absolute number of CD4+ CD20+ follicular helper T (CXCR5+, TFH) cells was detected in CLL patients compared to the control group (**Figures 3B**, **4B**). In turn, the percentage of CD4+ CD20+ TFH cells was increased in both MBL groups compared to controls (**Figure 3B**). Among, CD4+ CD20+ TFH cells, a significant increase in the percentage and absolute number of naïve cells was found in CLL compared to both controls and HC-MBL (**Figures 3D**, **4D**). In contrast, there was a decrease in the percentage of effector memory cells in CLL compared to controls. In all patient groups, the majority of CD4+CD20 TFH cells were Th1-like TFH cells (**Figure 3E**). Furthermore, a statistically significant increase in percentage and absolute number of Th1-like TFH cells was observed in HC-MBL, compared to controls while Th17-like TFH cells were significantly lower in HC-MBL vs LC-MBL (**Figures 3E**, **4E**).

CD4+ CD20+ Th17 cells showed similar frequencies among all groups (**Figures 3B**, **4B**). However, the majority of CD4+ CD20+ Th17 cells from both MBL groups were CCR5-. This is in contrast with CLL where we found a significant decrease in the percentage and absolute number of CD4+ CD20+ Th17 cells that did not express CCR5 (CCR6+ CCR5-) vs the controls (**Figures 3H**, **4H**).

Interestingly, a progressive increase in the percentage of CD4+ CD20+ Th1 cells was observed from controls to LC-MBL, HC-MBL and CLL, with statistically significant differences between HC-MBL and both controls and LC-MBL (**Figure 3B**). An increased percentage of CD4+ CD20+ Th1 cells within the effector memory compartment was observed in HC-MBL compared to LC-MBL, at the expense of naïve T cells (**Figure 3J**). In addition, an increased number of naïve and decreased counts of effector memory cells were found in CLL compared to HC-MBL (**Figure 3J**). We also observed a decrease in the absolute numbers of central memory cells in CLL compared to controls (**Figure 4J**).

As shown in **Figure 3C**, a statistically significant increase in the percentage of activated (CD25+) CD4+ CD20+ T cells was detected in LC-MBL, compared to controls and HC-MBL (**Figure 3C**). Among the studied functional subpopulations, no statistically significant differences were observed in the percentage of activated cells (**Figures 3F, I, K**). In absolute numbers, activated (CD25+) CD4+ CD20+ TFH cells were also increased in CLL compared to controls (**Figure 4F**). Meanwhile, among CD4+ CD20+ Th17 cells, a significant decrease of activated cells (CD25+) was observed in all patient groups vs the controls (**Figures 4C, I**). Among CD4+ CD20+ Th1 cells, no significant differences were found (**Figure 4K**).

#### 3.2.2 CD8+ CD20+ T cells

**Figure 5:**
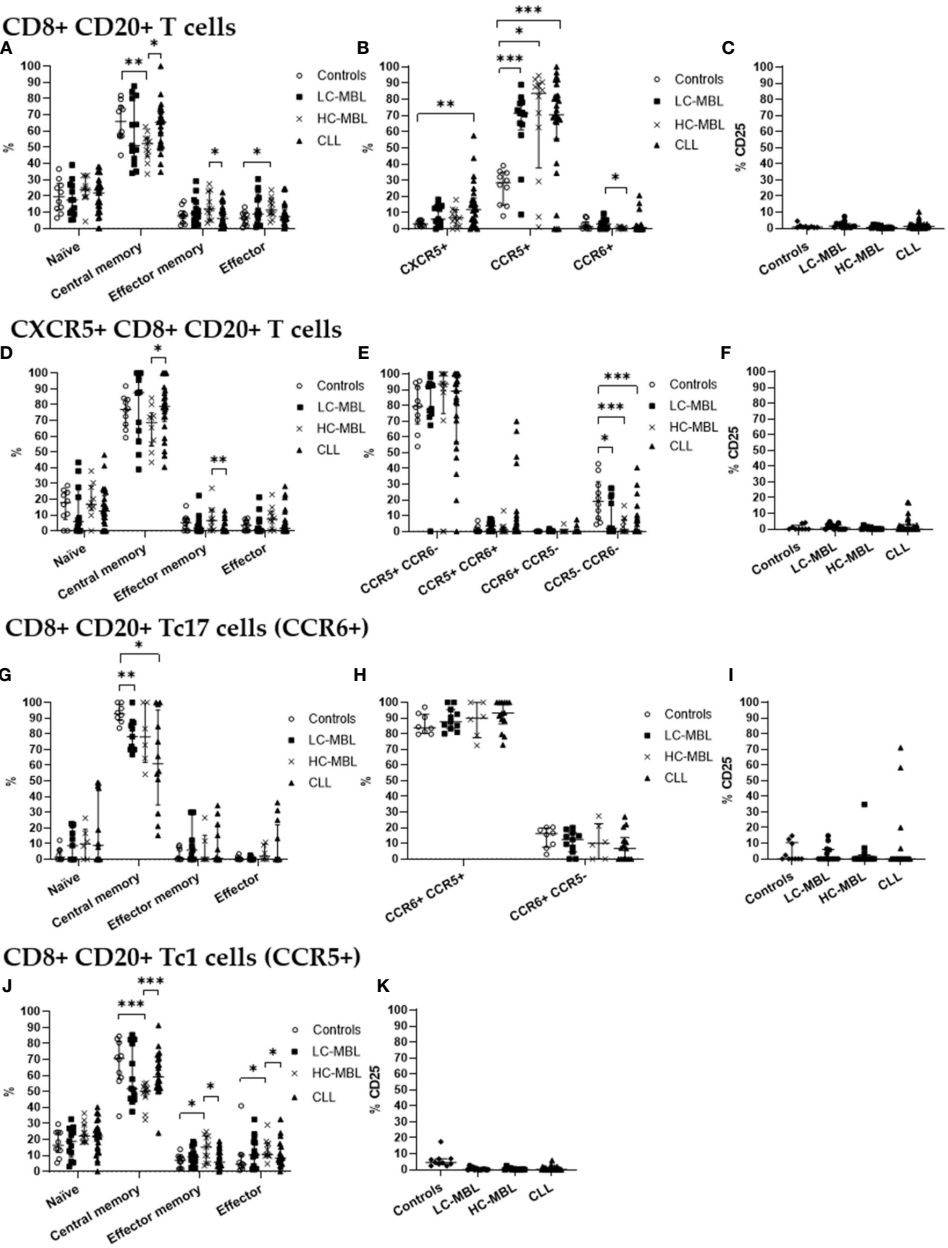
Phenotypic characterization of CD8+ CD20+ T cells and their subsets in blood of LC-MBL, HC-MBL and CLL vs controls. **(A-C)** Analysis of total CD8+ CD20+ T cells: **(A)** distribution within each different maturation-associated compartment of CD20+ T cells (naïve, central memory, effector memory and terminal effector); **(B)** percentage of CXCR5+ CD8+ CD20+ T cells, CD20+ Tc1 and CD20+ Tc17 cells; and **(C)** percentage of activated (CD25+) CD8+ CD20+ T cells. **(D-F)** Distribution of CD8+ CD20+ T cells expressing CXCR5 in **(D)** the different maturation-associated of T cell compartments (naïve, central memory, effector memory and terminal effector); **(E)** CXCR5+ CD8+ CD20+ T cell subsets (Tc1-like, Tc17-like, Tc1/17-like and CCR5- CCR6- T cells); and **(F)** percentage of activated (CD25+) CXCR5+ CD8+ CD20+ T cells. **(G-I)** CD20+ Tc17 cells: **(G)** frequency of cells in the different maturation-associated compartments of CD20+ T cells (naïve, central memory, effector memory and terminal effector; **(H)** percentage of CD20+ Tc17 cells CCR5+ or CCR5-; and **(I)** percentage of activated (CD25+) CD20+ Tc17 cells. **(J)** Frequency of CD20+ Tc1 cells within each different maturation-associated compartments of CD20+ T cells: naïve, central memory, effector memory and terminal effector. **(K)** Percentage of activated (CD25+) CD20+ Tc1 cells. Data expressed as median and interquartile range. *p < 0.05; **p < 0.01; ***p < 0.001. CLL, chronic lymphocytic leukemia; HC, high-count; LC, low-count; MBL, monoclonal B lymphocytosis.

**Figure 6:**
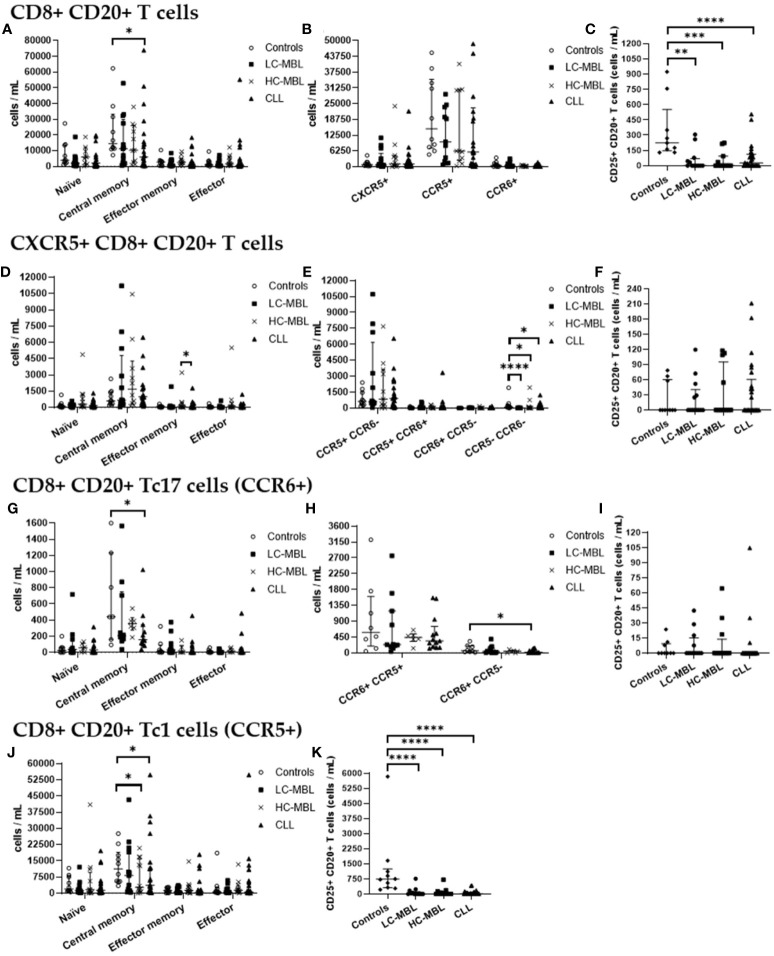
Absolute number of CD8+ CD20+ T cells and their subsets in blood of LC-MBL, HC-MBL and CLL vs controls, and absolute number of the phenotypically defined CD8+ CD20+ T cell subsets. **(A-C)** Analysis of total CD8+ CD20+ T cells: **(A)** distribution within each different maturation-associated compartments of CD20+ T cells (naïve, central memory, effector memory and terminal effector); **(B)** absolute number of CXCR5+ CD8+ CD20+ T cells, CD20+ Tc1 and CD20+ Tc17 cells; and **(C)** absolute number of activated (CD25+) CD8+ CD20+ T cells. **(D-F)** Distribution of CD8+ CD20+ T cells expressing CXCR5 in **(D)** the different maturation-associated T cell compartments (naïve, central memory, effector memory and terminal effector); and **(E)** CXCR5+ CD8+ CD20+ T cell subsets (Tc1-like, Tc17-like, Tc1/17-like and CCR5- CCR6- T cells); and **(F)** absolute number of activated (CD25+) CXCR5+ CD8+ CD20+ T cells. **(G-I)** Analysis of CD20+ Tc17 cells: **(G)** absolute number of cells in the different maturation-associated compartments of CD20+ T cells (naïve, central memory, effector memory and terminal effector; **(H)** absolute number of CD20+ Tc17 cells CCR5+ or CCR5-; and **(I)** absolute number of activated (CD25+) CD20+ Th17 cells. **(J)** Absolute number of CD20+ Tc1 cells within each different maturation-associated compartments of CD20+ T cells: naïve, central memory, effector memory and terminal effector. **(K)** Absolute number of activated (CD25+) CD20+ Tc1 cells. Data were expressed as the median with interquartile range. *p < 0.05; **p < 0.01; ***p < 0.001; ****p < 0.0001. CLL, Chronic Lymphocytic Leukemia; HC, high-count; LC, low-count; MBL, Monoclonal B Lymphocytosis.

Similarly to what has been described above for CD4+ CD20+ T cells, CD8+ CD20+ T cells, in healthy individuals, also showed a predominant central memory phenotype (**Figures 5A**, **6A**). This memory phenotype predominated among all functional subpopulations of CD8+ CD20+ T cells identified, including Tc1, Tc17 and CXCR5+ T cells (**Figures 5D, G, J**, **6D, G, J**). Furthermore, in healthy individuals, the majority of CD8+ CD20+ T cells expressing CXCR5 displayed a Tc1-like phenotype, while the majority of CD8+ CD20+ Tc17 cells also expressed CCR5 (**Figures 5E, H**, **6E, H**).

Though CD8+ CD20+ T cells from all the studied groups predominantly belonged to the central memory compartment, in HC-MBL a higher amount of CD8+ CD20+ T cells which had undergone differentiation into the effector memory and effector compartments (with a consequent decrease in central memory cells) was observed, compared to both the control group and CLL patients (**Figure 5A**). In addition, a significant decrease in the absolute number of central memory cells was also observed in CLL when compared to the control group (**Figure 6A**).

Regarding CD8+ CD20+ T cells an increased percentage of CXCR5+ cells was found in all patient groups compared to controls, but this increase was only significant in CLL (vs control group) (**Figure 5B**). Regarding the absolute values of CXCR5+ cells, no significant differences were observed (**Figure 6B**). Among, CD8+ CD20+ T cells expressing CXCR5, a significant increase in central memory cells, with a decreased percentage (and absolute number) of effector memory cells was found in CLL compared to HC-MBL (**Figures 5D**, **6D**). Of note, the majority of CD8+ CD20+ expressing CXCR5 displayed a Tc1-like phenotype in LC-MBL, HC-MBL and CLL patients (**Figure 5E**).

Among CD8+ CD20+ T cells, there was a statistically significant decrease in the number of CD8+ CD20+ Tc17 cells in HC-MBL compared to LC-MBL (**Figure 5B**). In addition, a decrease in central memory CD8+ CD20+ Tc17 cells was observed in LC-MBL and CLL, compared to the control group (**Figures 5G**, **6G**). Interestingly, the majority of CD8+ CD20+ Tc17 cells from both MBL and CLL patients, also expressed CCR5 (**Figure 5H**).

A statistically significant increase in CD8+ CD20+ Tc1 cells was found in all patient groups compared to the control group (**Figure 5B**). In HC-MBL, the percentage and absolute number of CD20+ Tc1 cells within the central memory compartment decreased, while their percentage in the effector memory and effector compartments increased, compared to both the control group and CLL (**Figures 5J**, **6J**).

Finally, considering the percentage of activated CD8+ CD20+ T cells, no significant differences were found between the study groups (**Figures 5C, F, I, K**). Considering the absolute number of CD8+ CD20+ T cells, a statistically significant decrease of activated (CD25+) cells was observed in CLL compared to controls, particularly among CD8+ CD20+ Tc1 cells (**Figures 6C, K**). Among CXCR5+ CD8+ CD20+ T cells and CD8+ CD20+ Tc17 cells, no significant differences were observed (**Figures 6F, I**).

### 3.3 TCR Vβ repertoire of CD4+ CD20+, CD4+ CD20-, CD8+ CD20+ and CD8+ CD20- T cells in blood of CLL

**Figure 7:**
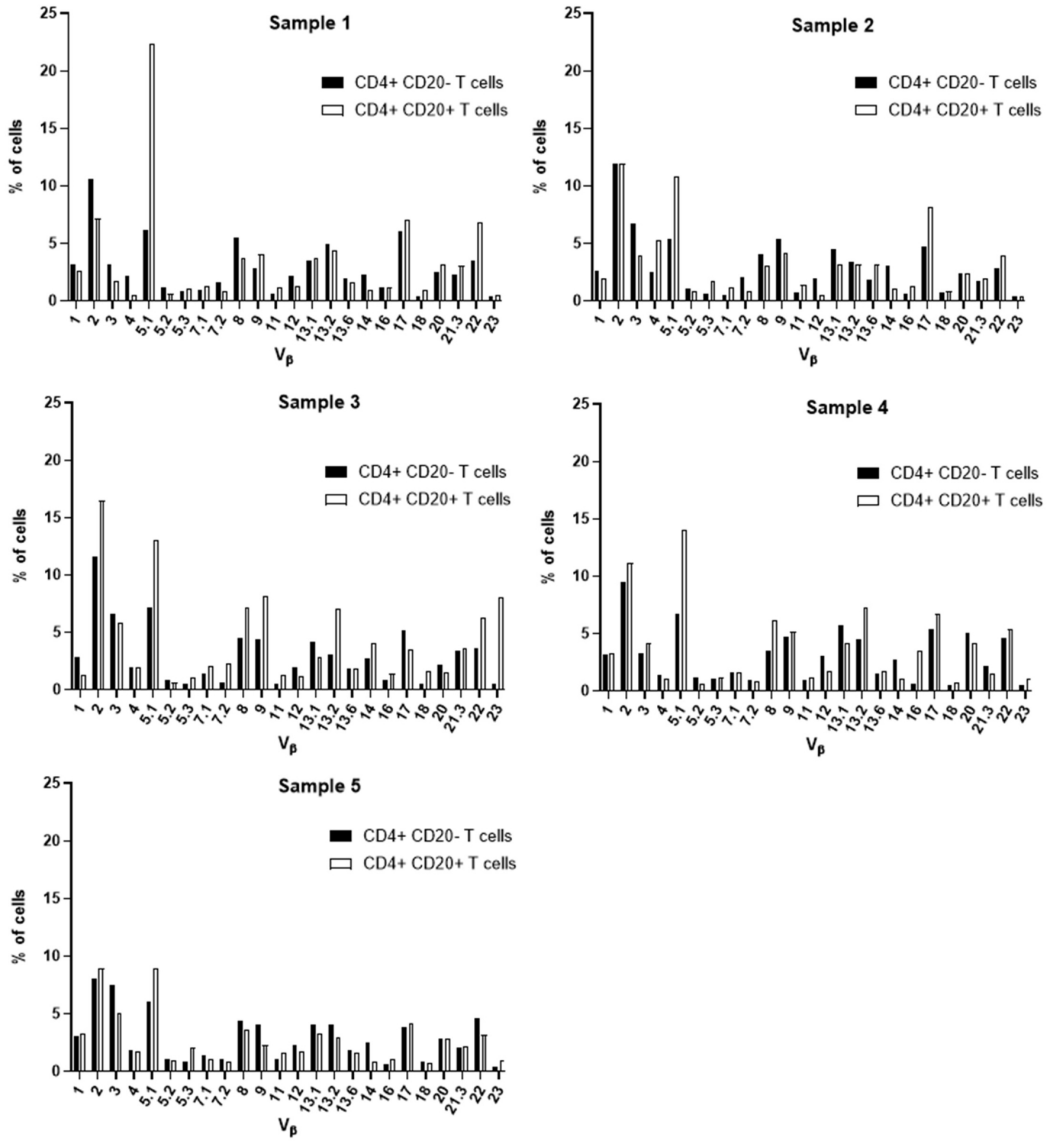
TCR Vβ repertoire usage in CD4+ T cells from peripheral blood of chronic lymphocytic leukemia patients: comparison between CD4+ CD20+ T cells and CD4+ CD20- T cells. Results for samples 1, 2, 3, 4 and 5.

**Figure 8:**
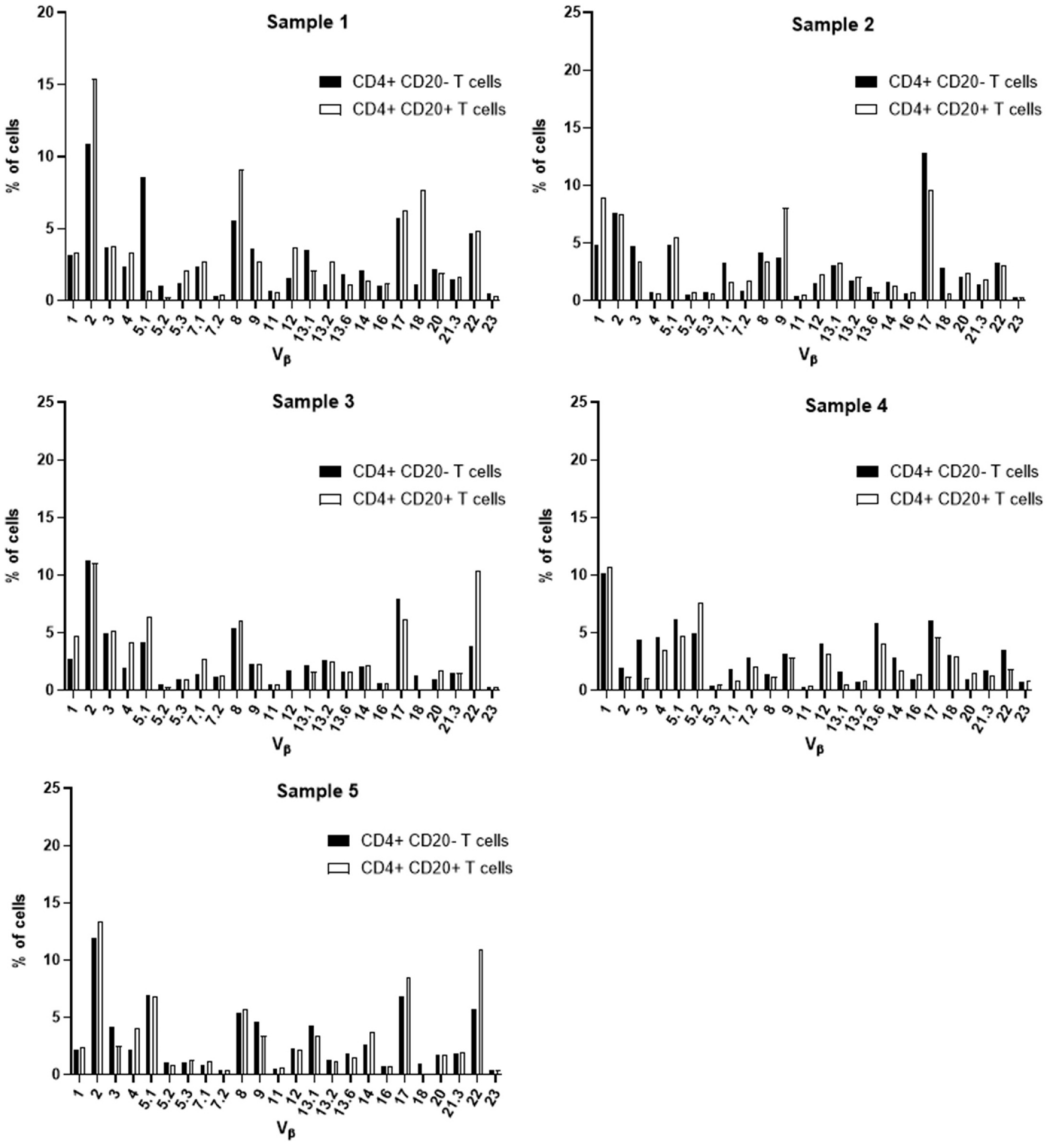
TCR Vβ repertoire usage in CD4+ T cells from peripheral blood of healthy controls: comparison between CD4+ CD20+ T cells and CD4+ CD20- T cells. Results shown for samples 1, 2, 3, 4 and 5.

**Figure 9:**
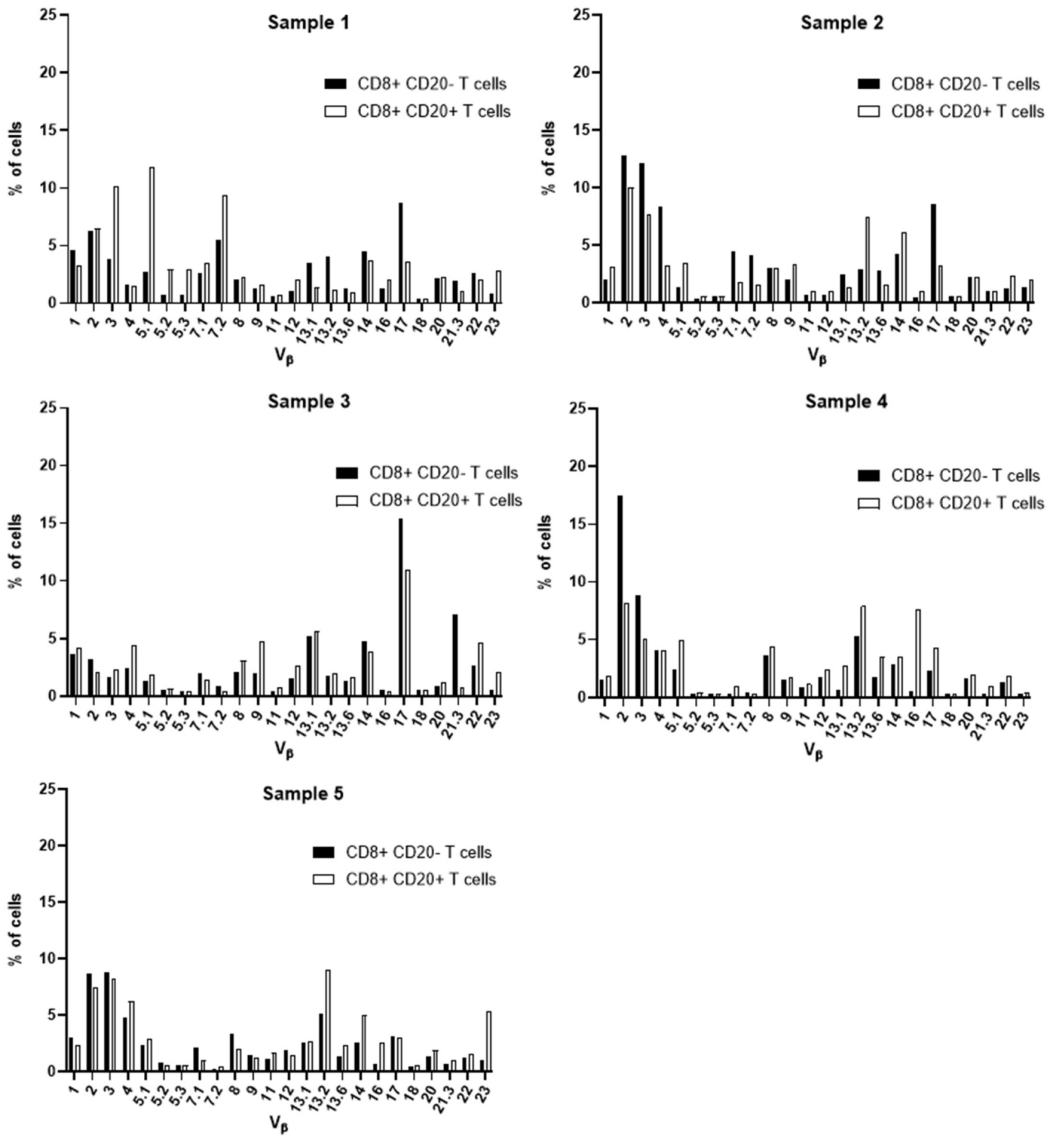
TCR Vβ repertoire usage in CD8+ T cells from peripheral blood of chronic lymphocytic leukemia patients: comparison between CD8+ CD20+ T cells and CD8+ CD20- T cells. Results for samples 1, 2, 3, 4 and 5.

**Figure 10:**
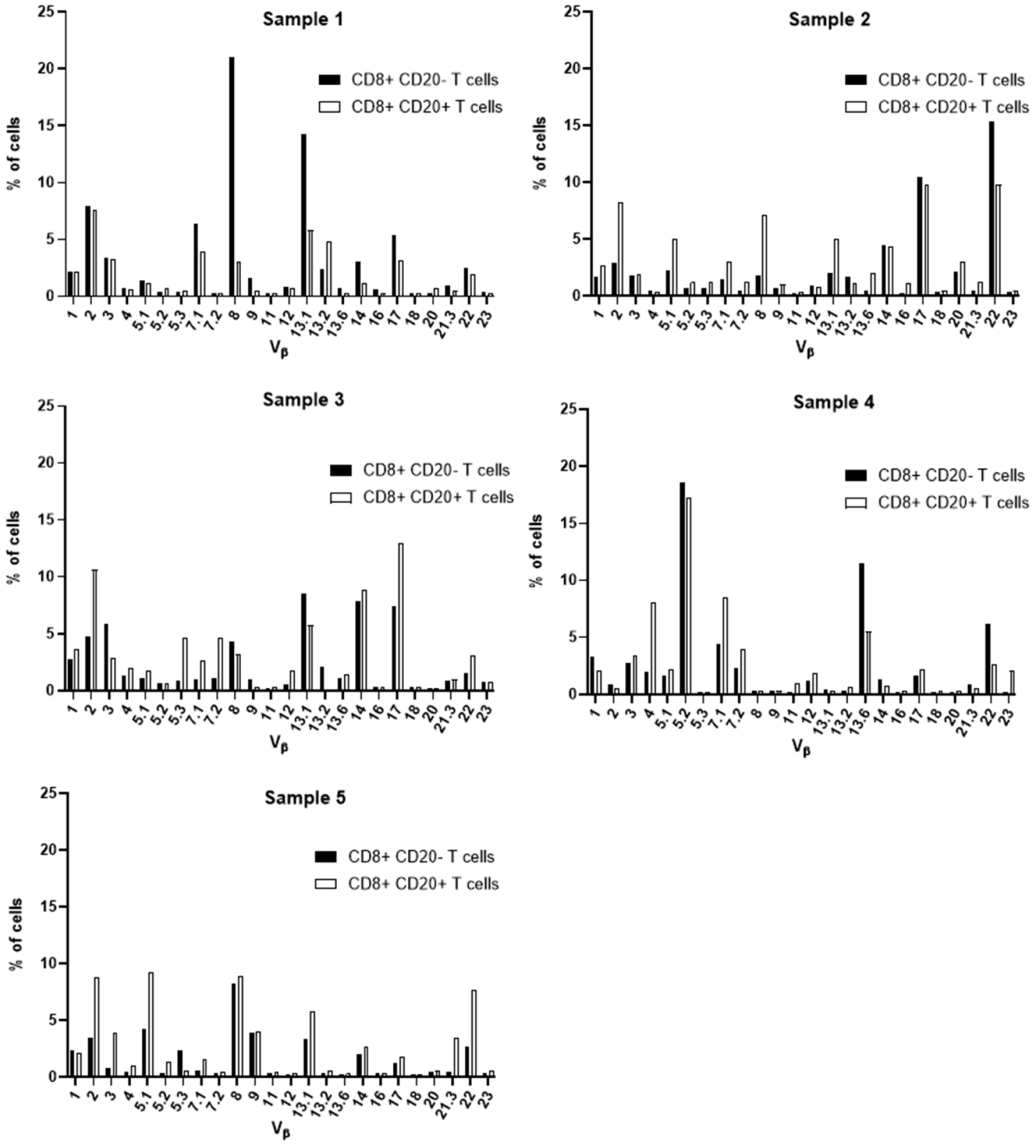
TCR Vβ repertoire usage in CD8+ T cells from peripheral blood of healthy controls: comparison between CD8+ CD20+ T cells and CD8+ CD20- T cells. Results shown for samples 1, 2, 3, 4 and 5.

Aiming at the identify potential differences in the TCR Vβ repertoire of CD20- T cells and CD20+ T cells, we analyzed the expression of 24 families of the variable region of the T cell receptor β chain (TCR-Vβ) (**Figures 7**–**10**). Compared to CD20- CD4+ T cells, CD4+ CD20+ T lymphocytes showed a predominant expansion (p<0.05) of T cells expressing the Vβ 5.1 family in all CLL patients (**Figure 7**). The median and interquartile range of the percentage of CD4+ T cells for the Vβ 5.1 family was 6.2% ± 1.2% in CD4+ CD20- cells versus 13% ± 8.3%, among CD4+ CD20+ positive T cells. Of note, this expansion was not observed in any subject from the control group (**Figure 8**). In addition to the expansion of CD4+ CD20+ T cells expressing the Vβ 5.1 family compared to CD4+ CD20- T cells, it was found that this expansion (p<0.05) also occurs among CLL patients compared to healthy individuals within CD4+ CD20+ T cells (**Supplementary Figure 4**). The median and interquartile range of the percentage of CD4+ CD20+ T cells for the Vβ 5.1 family was 5.5% ± 3.9% in healthy subjects versus 13% ± 8.3% in CLL patients. In contrast, no clear expansion of any TCR Vβ family was observed within CD8+ CD20+ T cells compared to CD8+ CD20- T cells (**Figures 9**, **10**).

Furthermore, no clear expansion of any TCR Vβ family was observed when comparing the same population between CLL patients and healthy subjects (CD8+ CD20+ T cells in CLL versus healthy subjects (**Supplementary Figure 5**).

### 3.4 IGHV and TP53 gene mutational status in HC-MBL and CLL

The search for mutations in the TP53 gene by NGS, identified mutations in 2/27 cases of CLL (7%) but none of 12 HC-MBL cases showed this mutation (**Table 3**).

**Table 3:** Percentage of CLL and MBL cases mutated for the TP53 gene and for the IGHV gene.

Molecular Study	HC-MBL (n = 12)	CLL (n = 27)	*P* value
**TP53 gene** Mutated	0/12 (0%)	2/27 (7%)	*P* > 0.99
**IGHV gene SHM status** Mutated	8/12 (67%)	15/27 (56%)	*P* = 0.73

CLL, chronic lymphocytic leukemia; HC, high-count; IGHV, immunoglobulin heavy chain variable region; LC, low-count; MBL, monoclonal B lymphocytosis; SHM, somatic hypermutation. Results expressed as number of cases with IGHV mutated and TP53 mutated genes from all cases analyzed in the corresponding group (and their respective percentage).

Determination of IGHV mutational status revealed 8/12(67%) and 15/27 (56%) mutated HC-MBL and CLL cases, respectively, based on a cut-off of 98% identity to the germline gene (**Table 3**).

Analysis of IGHV gene rearrangements, performed for each purified population of clonal B cells in HC-MBL and CLL (**Table 4**), revealed that the IGHV1-69, IGHV3-23, IGHV3-30, and IGHV4-34 genes were the most used and represented 49% of all HC-MBL and CLL cases. Although HC-MBL shared these same rearrangements found in CLL, the IGHV3-30 and IGHV3-33 genes were only detected in CLL (26%) (**Table 4**). In turn, IGHV1-69 was the most used gene among the unmutated cases (50% of IGHV unmutated HC-MBL and 25% of IGHV unmutated CLL cases), whereas IGHV3-23 and IGHV4-34 gene predominated among the IGHV mutated cases (63% of HC-MBL vs 34% of CLL mutated cases) (**Table 4**).

**Table 4:** IGHV gene usage by HC-MBL and CLL cases, and its distribution by mutated and unmutated IGHV gene.

IGHV gene	HC-MBL			CLL		
	IGHV Mutated	IGHV Unmutated	Total	IGHV Mutated	IGHV Unmutated	Total
IGHV1-3	0/8 (0%)	0/4 (0%)	0/12 (0%)	0/15 (0%)	1/12 (8%)	1/27 (4%)
IGHV1-18	0/8 (0%)	0/4 (0%)	0/12 (0%)	0/15 (0%)	1/12 (8%)	1/27 (4%)
IGHV1-60	0/8 (0%)	1/4 (25%)	1/12 (8%)	0/15 (0%)	0/12 (0%)	0/27 (0%)
IGHV1-69	0/8 (0%)	2/4 (50%)	2/12 (17%)	0/15 (0%)	3/12 (25%)	3/27 (11%)
IGHV2-5	1/8 (13%)	0/4 (0%)	1/12 (8%)	0/15 (0%)	0/12 (0%)	0/27 (0%)
IGHV3-7	1/8 (13%)	0/4 (0%)	1/12 (8%)	1/15 (7%)	0/12 (0%)	1/27 (4%)
IGHV3-9	0/8 (0%)	0/4 (0%)	0/12 (0%)	0/15 (0%)	1/12 (8%)	1/27 (4%)
IGHV3-15	0/8 (0%)	0/4 (0%)	0/12 (0%)	1/15 (7%)	0/12 (0%)	1/27 (4%)
IGHV3-21	0/8 (0%)	0/4 (0%)	0/12 (0%)	0/15 (0%)	1/12 (8%)	1/27 (4%)
IGHV3-23	2/8 (25%)	0/4 (0%)	2/12 (17%)	1/15 (7%)	0/12 (0%)	1/27 (4%)
IGHV3-30	0/8 (0%)	0/4 (0%)	0/12 (0%)	2/15 (13%)	2/12 (17%)	4/27 (15%)
IGHV3-33	0/8 (0%)	0/4 (0%)	0/12 (0%)	2/15 (13%)	1/12 (8%)	3/27 (11%)
IGHV3-48	0/8 (0%)	0/4 (0%)	0/12 (0%)	1/15 (7%)	0/12 (0%)	1/27 (4%)
IGHV3-53	1/8 (13%)	0/4 (0%)	1/12 (8%)	0/15 (0%)	0/12 (0%)	0/27 (0%)
IGHV3-74	0/8 (0%)	0/4 (0%)	0/12 (0%)	1/15 (7%)	0/12 (0%)	1/27 (4%)
IGHV4-4	0/8 (0%)	0/4 (0%)	0/12 (0%)	0/15 (0%)	1/12 (8%)	1/27 (4%)
IGHV4-34	3/8 (38%)	0/4 (0%)	3/12 (25%)	4/15 (27%)	0/12 (0%)	4/27 (15%)
IGHV4-61	0/8 (0%)	0/4 (0%)	0/12 (0%)	1/15 (7%)	0/12 (0%)	1/27 (4%)
IGHV5-10	0/8 (0%)	1/4 (25%)	1/12 (8%)	0/15 (0%)	0/12 (0%)	0/27 (0%)
IGHV5-51	0/8 (0%)	0/4 (0%)	0/12 (0%)	0/15 (0%)	1/12 (8%)	1/27 (4%)
IGHV6-1	0/8 (0%)	0/4 (0%)	0/12 (0%)	1/15 (7%)	0/12 (0%)	1/27 (4%)

IGHV, immunoglobulin heavy chain variable region. Results expressed as a number and percentage of HC-MBL and CLL cases for each IGHV gene detected. No statistically significant differences were found between HC-MBL vs CLL.

**Figure 11:**
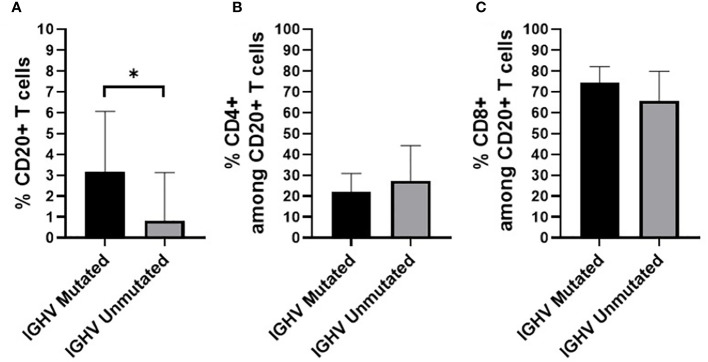
Percentage of **(A)** CD20+ T cells (in whole blood) **(B)** CD4+ T cells and **(C)** CD8+ T cells, in HC-MBL and CLL patients considered together and distributed according to the presence of a mutated vs unmutated IGHV B-cell receptor. Data expressed as mean and standard deviation. *p < 0.05. IGHV, immunoglobulin heavy chain variable region.

Finally, we also evaluated whether the percentage of CD20+ T cells, and their distribution among the CD4+ and CD8+ T cell populations, varied according to the IGHV mutational status of both HC-MBL and CLL (**Figure 11**). Overall, statistically significant decrease in the frequency of CD20+ T cells was observed among CLL and HC-MBL cases with unmutated vs mutated IGHV gene profile (**Figure 11**).

## 4 Discussion

Cooperation between B cells and T cells is crucial for a robust and efficient immune response, as these cells are the cornerstone of adaptive immunity and, together with NK cells, critical cells for immune surveillance. At present, it is well-established that B cell neoplasms are characterized by a prominent and broad dysregulation of the immune response. Indeed, evidences have been described in CLL and CLL-like MBL that confirm a marked disruption of the normal immune homeostasis due to the interaction between pathological B cells (CD5+) and T cells (3, 11, 28).

In recent years, CD20+ T cells have been described in the literature to be involved in the pathophysiology of autoimmune diseases and cancer, where numerical, phenotypic, and functional alterations have been detected in this subset of T cells (18–24), fully in line with our findings in this study for CD20+ T cells in subjects presenting LC-MBL, HC-MBL, and CLL patients.

Despite the origin of CD20+ T cells is poorly understood, several studies have described trogocytosis as the process responsible for the transfer of the CD20 molecule from B cells to T cells as a result of the close interaction between both groups of cells. Based on this hypothesis, trogocytosis would possibly occur when B cells activate T cells during the immune response (22, 29). Here, we found decreased numbers of CD20+ T cells in peripheral blood of LC-MBL and CLL patients, even when the percentage of CD20+ T cells in blood was calculated after excluding the neoplastic B cells. These findings suggest that trogocytosis might not explain the decrease in the frequency of CD20+ T cells in MBL and CLL, when compared to non-MBL healthy individuals, as in both conditions an accumulation of B cells exists, which could facilitate the contact and interaction with T cells. However, neoplastic B cells from MBL and CLL display low expression of CD20, which corresponds to a lower amount of CD20 protein per cell (30, 31), and the accumulation of neoplastic B cells in these conditions may hamper the interaction between T cells and normal B cells in CLL patients and, to a lesser extent, in MBL.

Among all T cell subsets, CD20+ T cells were more represented in the CD8+ T cell compartment in our cohort, in line with previous studies (15, 17, 18), in the absence of significant differences between the studied and control groups. However, when we investigated the different functional compartments of CD4+ CD20+ and CD8+ CD20+ T cells, the majority of them presented a memory (central and effector) Th1 or Tc1 phenotype (15, 17, 18, 32). Cytotoxic CD8+ T cells play an important role in antitumor immunity since, together with NK cells, they are the main effector cells responsible for eliminating neoplastic cells (33). Furthermore, the predominantly memory phenotype suggests that the majority of CD20+ T cells are antigen experienced cells (15, 34).

An overall decrease in naïve CD4+ T cells, accompanied by an increase in the memory cell compartments, had been reported in CLL compared to healthy individuals (3, 12, 35). Overall, we could not confirm these alterations within the CD4+ CD20+ T cell compartment, as no differences between CLL and healthy controls were observed, despite an increase in CD4+ CD20+ central memory cells accompanied by a decrease in effector memory cells was found in CLL vs HC-MBL. However, an increase in CD4+ CD20+ TFH cells compared to healthy individuals was observed in patients with LC-MBL, HC-MBL and CLL. Of note, these cells mainly displayed a Th1-like TFH phenotype. These results are consistent, at least in part, with previous studies which described an increased frequency of TFH cells in CLL (36, 37), but contradicted the data reported by Wu et al., 2021 (38), who did not observe an increase in TFH cells in MBL. Overall, these results also suggest that the expansion of CD20+ TFH cells parallels the expansion of the B-CLL clone, which would indirectly support the notion that TFH cells might provide survival and proliferation signals to pathological B cells in both MBL and CLL (38, 39). In our study, an expansion of CD4+ CD20+ Th1 cells was also observed in the blood of patients with HC-MBL. Th1 cells have been shown to mediate the immune response against tumor cells through secretion of IFN-γ, and in CLL, IFN-γ is associated with the inhibition of apoptosis of B-CLL clones (40, 41). An increased percentage of activated (CD25+) CD20+ T cells, predominantly within the CD4+ T cell compartment, has been described by Roessner & Seiffert, 2020 (10) in CLL; such activated T cells in patients with CLL contribute to the proliferation and survival of B-CLL cell clones (10, 42). Here, we detected an increased percentage of activated (CD25+) CD4+ CD20+ T cells from LC-MBL patients, but not in HC-MBL or CLL.

When we analyzed the distribution of CD8+ CD20+ T cells according to the maturation-associated T cell compartments of naïve, central memory, effector memory, and terminal effector cells in the control group, we found most cells to be memory cells. As for other functional T cell subsets, CD8+ CD20+ T cells also exhibited here a predominantly Tc1 phenotype (18, 32). However, phenotypic analysis of CD8+ CD20+ T cells revealed an increased percentage of CXCR5+ cells in CLL compared to the control group, and an expansion of CD8+ CD20+ Tc1 cells that was consistent across all patient groups. The cytotoxic function of CD8+ T cells, particularly of Tc1 cells, has been associated with a key role in mediating T cell antitumoral responses (18, 24, 43, 44).

Several alterations of different T cell compartments have been described as relevant factors in the dysregulation of the immune response in the tumor microenvironment in CLL, resulting in an impaired antitumor immunity (3, 12). In CLL, this has been associated with a restricted usage in the T cell receptor (TCR) gene repertoire (3, 13, 45). Here, we further characterized the TCR-Vβ gene family usage of CD4+ CD20- T and CD8+ CD20- T cells compared to their CD4+ CD20+ T and CD8+ CD20+ T cell counterparts, respectively. Of note, among CD4+ CD20+ T cells from CLL patients, a relative expansion of T cells expressing the TCR Vβ 5.1 gene family was observed in all CLL patients analyzed. Interestingly, bystander expansions of TCR Vβ 5.1 have been reported in the literature for CLL, but its meaning remains to be understood (13, 45, 46).

Altogether, the above findings suggest that in CLL and its precursor states of MBL, the expansion of neoplastic B cells is accompanied by phenotypic and functional changes in the T cell compartment of CD20+ T cells. The abnormal distribution of T cell subpopulations, and in particular the decreased number of CD20+ T cells with a cytotoxic phenotype, might contribute to the dysregulation of the immune system in MBL and CLL, potentially reflecting a decreased antitumor activity and the inability of T cells to complete an effective immune response against neoplastic B cells which may acquire additional genetic abnormalities (47–51). Determination of the mutational status of the IGHV gene revealed that about half of all CLL cases displayed mutated IGHV gene, at a (slightly) lower percentage than found in HC-MBL, although these differences did not reach statistical significance, in line with previous studies (5, 7, 52). Analysis of the specific IGHV gene usage by purified neoplastic B cells showed that HC-MBL shares the IGHV3-23, IGHV1-69, and IGHV4-34 gene repertoire with CLL, although a greater prevalence of the IGHV4-34 and IGHV3-23 gene in HC-MBL was observed, particularly among the mutated clones, in line with previous studies (7, 9, 53, 54). In contrast, IGHV1-69 gene, that was typical in CLL, has been associated with an unmutated profile and adverse features in CLL (3, 5, 7, 49, 52). Of note, greater CD20+ T cells numbers were found in this study, associated with mutated IGHV gene, which suggests a protective role of CD20+ T cells for both in MBL and CLL. Further studies, in larger cohorts of MBL and CLL patients, are needed in order to determine whether CD20+ T cells are in fact associated with a greater risk of progression of MBL to CLL.

## 5 Conclusion

In summary, here we describe a decreased in CD20+ T cells in blood of both MBL and CLL patients consistent with a predominantly Tc1 memory cell phenotype, particularly among IGHV unmutated cases. These results suggest that CD20+ T cells might have a pathogenic role in MBL and CLL as tumor-suppressive T cell population, which decreases on frequency in parallel to the expansion of the neoplastic B cells. Further studies in larger cohorts of patients with long follow-up are needed to clarify whether CD20+ T cells might be in fact associated with the risk of progression from MBL to CLL.

## Data availability statement

The data presented in the study are deposited in the https://www.ebi.ac.uk/eva/?eva-study=PRJEB73349, accession number PRJEB73349.

## Ethics statement

The studies involving humans were approved by Ethics Committee of Faculty of Medicine, University of Coimbra. The studies were conducted in accordance with the local legislation and institutional requirements. The participants provided their written informed consent to participate in this study. Written informed consent was obtained from the individual(s) for the publication of any potentially identifiable images or data included in this article.

## Author contributions

CR: Data curation, Formal analysis, Investigation, Methodology, Validation, Visualization, Writing – original draft, Writing – review & editing. PL: Formal analysis, Validation, Visualization, Writing – review & editing. APi: Validation, Writing – review & editing. IS: Investigation, Writing – review & editing. SS: Investigation, Writing – review & editing. MC: Investigation, Methodology, Writing – review & editing. ACO: Investigation, Writing – review & editing. AS: Investigation, Writing – review & editing. ID: Investigation, Resources, Writing – review & editing. HS: Resources, Writing – review & editing. MU: Investigation, Resources, Writing – review & editing. AS: Resources, Writing – review & editing. CG: Resources, Writing – review & editing. MD: Supervision, Writing – review & editing. JA: Writing – review & editing. IC: Writing – review & editing. AO: Visualization, Writing – review & editing. APa: Conceptualization, Formal analysis, Funding acquisition, Methodology, Project administration, Resources, Supervision, Validation, Visualization, Writing – review & editing.
