# Supplementary material for: CD20+ T cells in monoclonal B cell lymphocytosis and chronic lymphocytic leukemia: frequency, phenotype and association with disease progression
Source: Front Oncol. 2024 Mar 28;14:1380648. doi: 10.3389/fonc.2024.1380648 (PMC11007165; doi:10.3389/fonc.2024.1380648)
Supplement: Supplementary file 1 [file Image_1.pdf]

frontiers

Supplementary Material

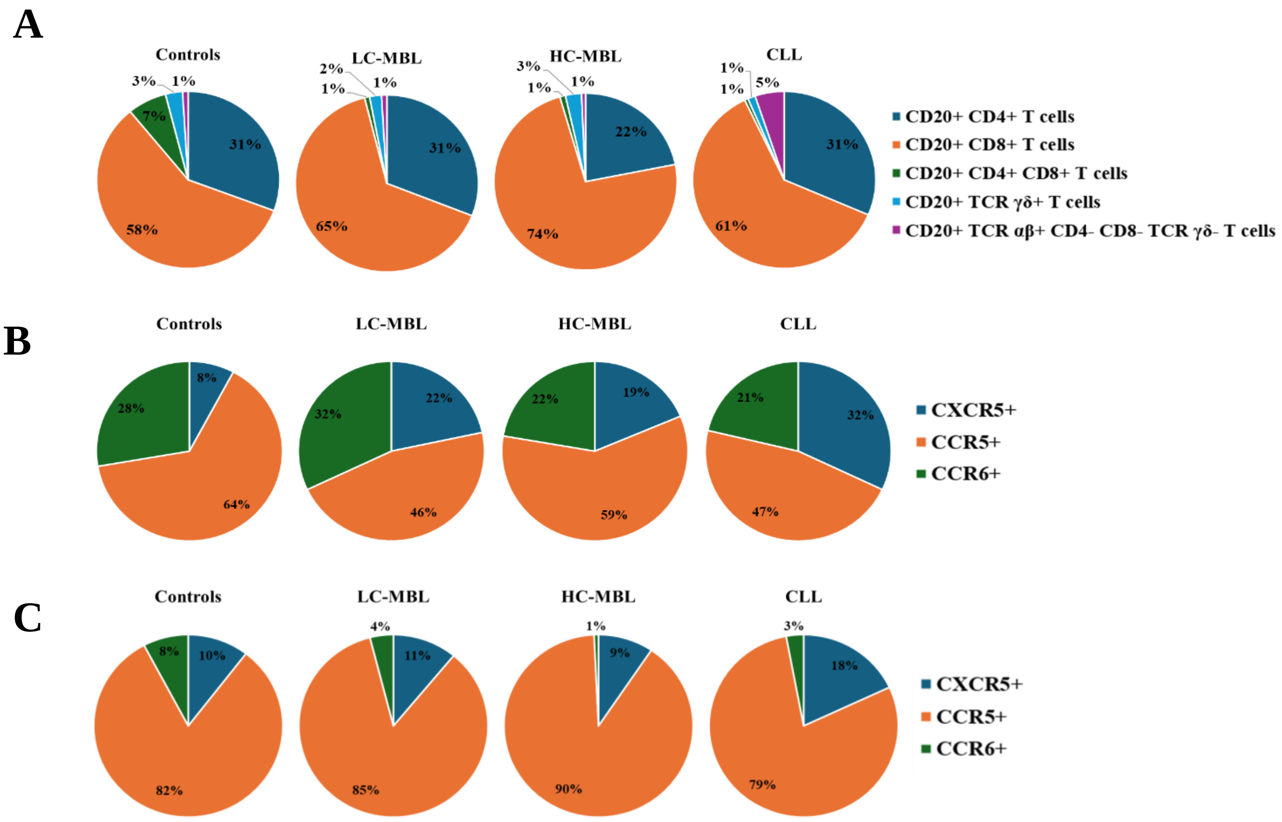

Supplementary Figure 1. Distribution of CD20+ T cells across the different subpopulations studied. **(A)** Analysis of distribution in the CD4+, CD8+, CD4+ CD8+, TCR γδ+ T cells and TCR αβ+ CD4- CD8- TCR γδ- T cells. **(B)** Analysis of functional subpopulations within CD4+ CD20+ T cells. **(C)** Analysis of functional subpopulations within CD8+ CD20+ T cells. CLL, chronic lymphocytic leukemia; HC, high-count; LC, low-count; MBL, monoclonal B lymphocytosis.
