# Supplementary material for: CD20+ T cells in monoclonal B cell lymphocytosis and chronic lymphocytic leukemia: frequency, phenotype and association with disease progression
Source: Front Oncol. 2024 Mar 28;14:1380648. doi: 10.3389/fonc.2024.1380648 (PMC11007165; doi:10.3389/fonc.2024.1380648)
Supplement: Supplementary file 3 [file Image_3.pdf]

Supplementary Material

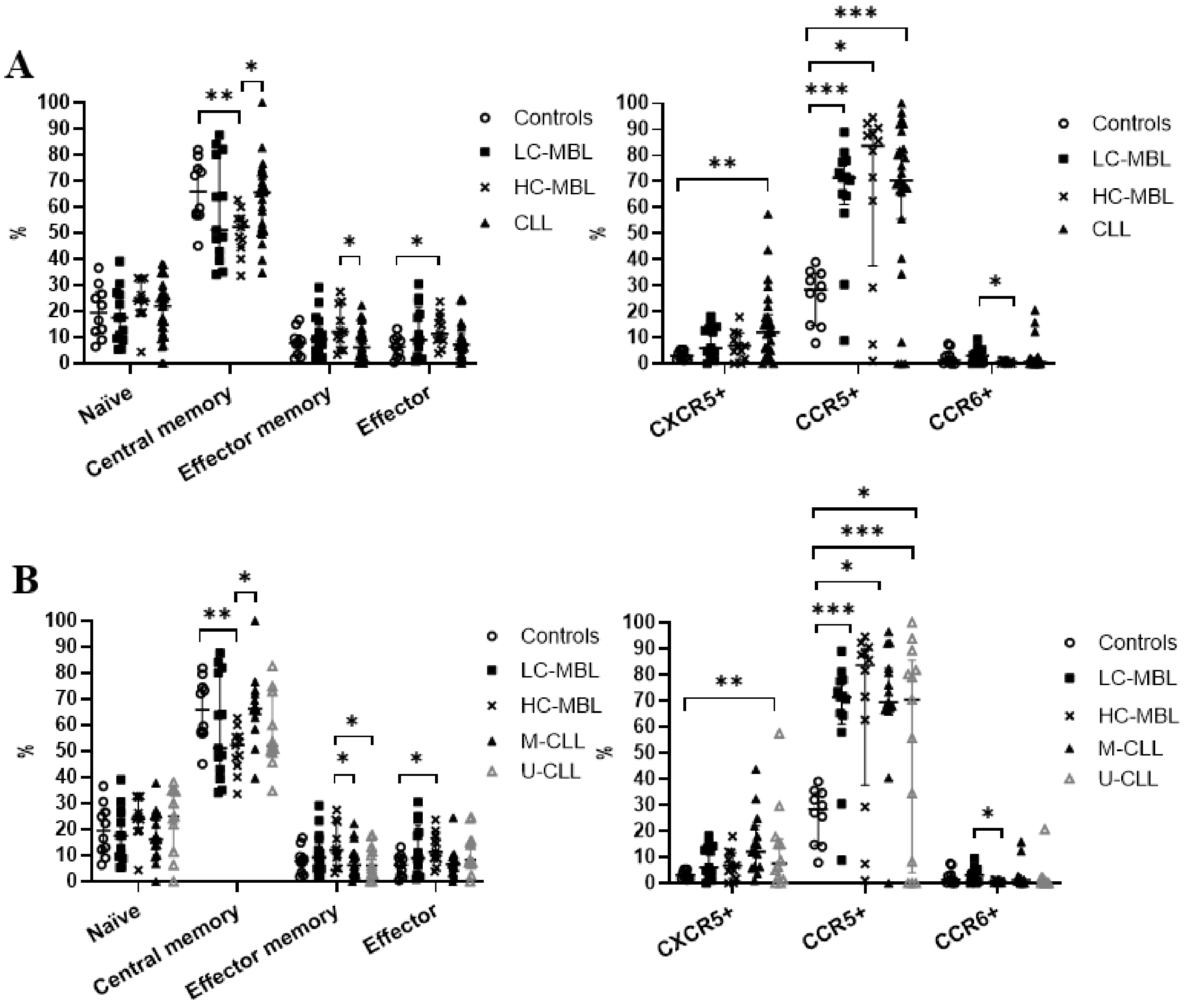

Supplementary Figure 3. (A) Phenotypic characterization of CD8+ CD20+ T cells in blood of LC-MBL, HC-MBL and CLL vs controls. **(B)** Analysis of total CD8+ CD20+ T cells in blood of LC-MBL, HC-MBL CLL with unmutated IGHV gene (U-CLL) and CLL with mutated IGHV gene (M-CLL) vs controls. Data expressed as median and interquartile range. *p < 0.05; **p < 0.01; *** p < 0.001. CLL, chronic lymphocytic leukemia; HC, high-count; LC, low-count; MBL, monoclonal B lymphocytosis.
