# Supplementary material for: CD20+ T cells in monoclonal B cell lymphocytosis and chronic lymphocytic leukemia: frequency, phenotype and association with disease progression
Source: Front Oncol. 2024 Mar 28;14:1380648. doi: 10.3389/fonc.2024.1380648 (PMC11007165; doi:10.3389/fonc.2024.1380648)
Supplement: Supplementary file 4 [file Image_4.pdf]

Supplementary Material

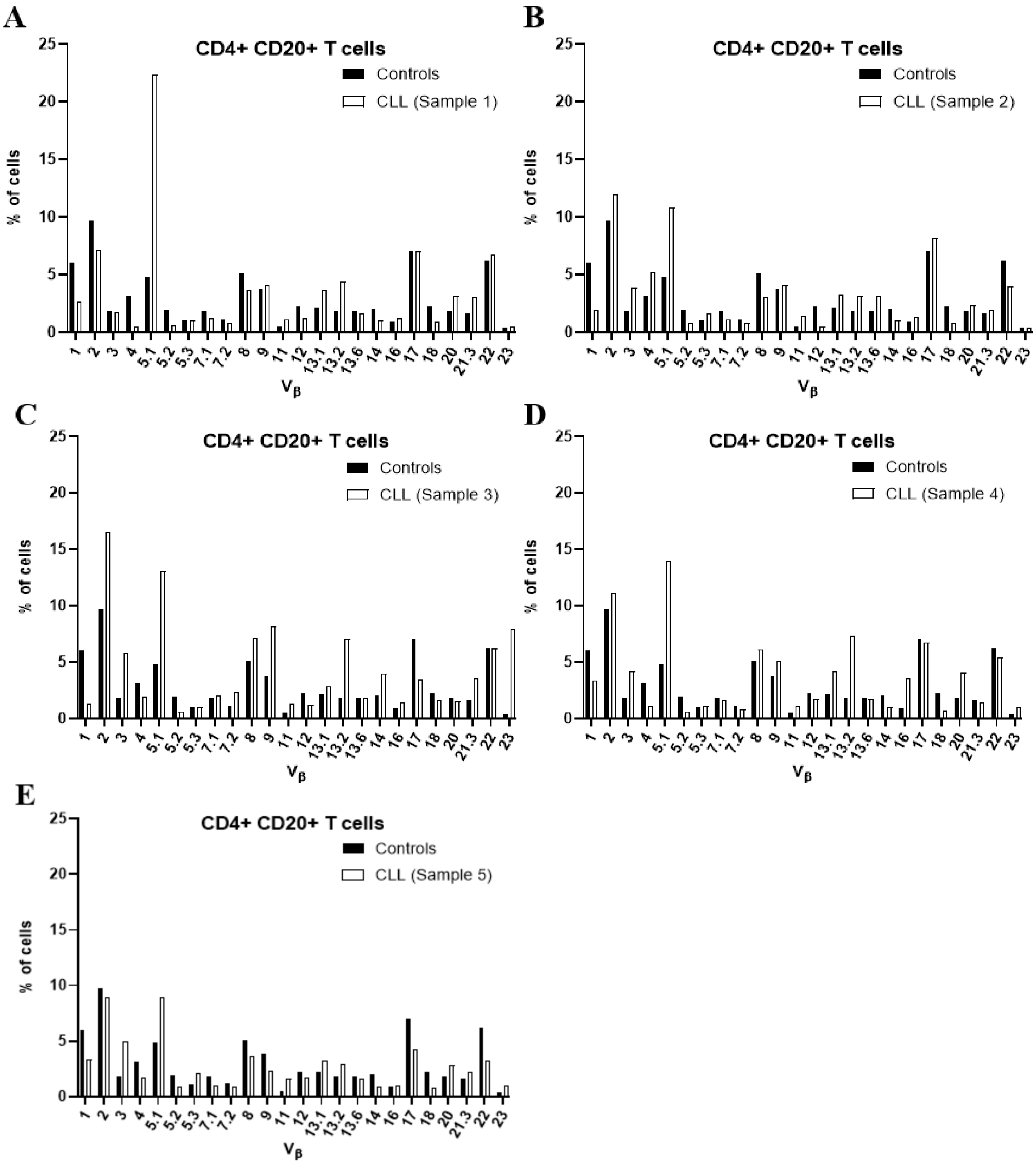

Supplementary Figure 4. TCR Vβ repertoire usage of five representative CLL patients compared with the mean control values for CD4+ CD20+ T cells. Results for **(A)** CLL (Sample 1); **(B)** CLL (Sample 2); **(C)** CLL (Sample 3); **(D)** CLL (Sample 4); **(E)** CLL (Sample 5).
